# Supplementary material for: Global rewiring of cellular metabolism renders Saccharomyces cerevisiae Crabtree negative
Source: Nat Commun. 2018 Aug 3;9:3059. doi: 10.1038/s41467-018-05409-9 (PMC6076296; doi:10.1038/s41467-018-05409-9)
Supplement: Supplementary file 1 — Supplementary Information [file 41467_2018_5409_MOESM1_ESM.pdf]

Global rewiring of cellular metabolism renders *Saccharomyces cerevisiae* Crabtree-negative

Dai *et al.*

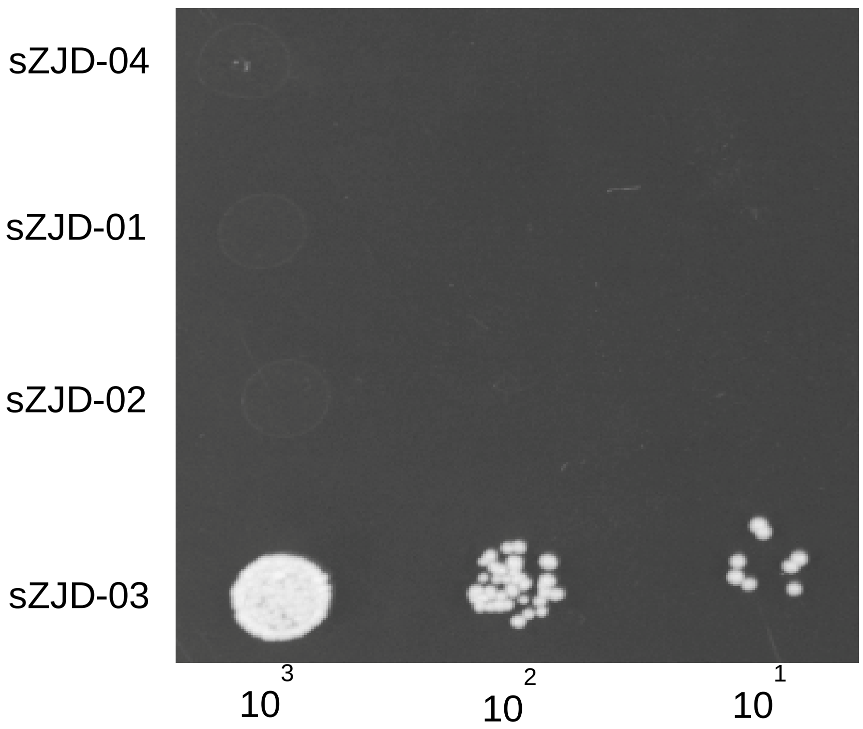

Supplementary Figure 1. Serial dilution of the different strains were grown on synthetic medium agar plates with 20 g l^{-1} glucose. sZJD-01: strain expressing PO gene from *S. pneumoniae* and PTA gene from *S. enterica*. sZJD-02: strain expressing PO gene from *L. plantarum* and PTA gene from *S. enterica*. sZJD-03: strain expressing PO gene from *A. viridans* and PTA gene from *S. enterica*. sZJD-04: control strain.

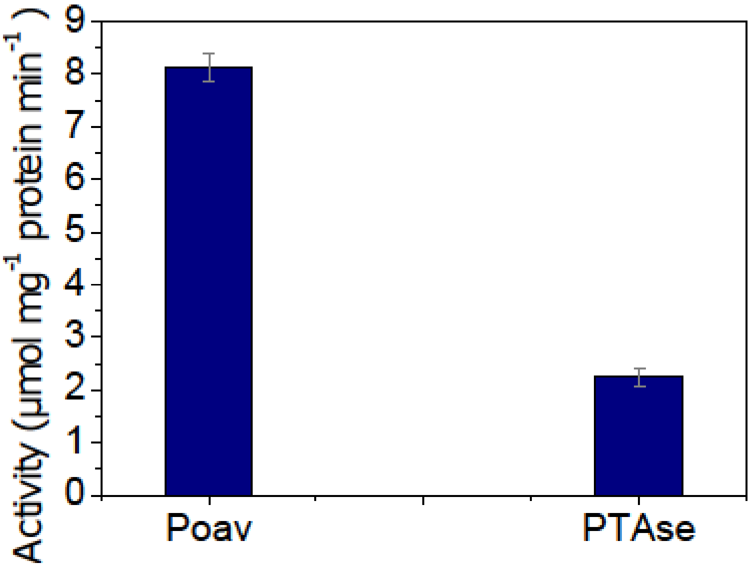

Supplementary Figure 2. Enzyme activity of pyruvate oxidase and phosphotransacetylase in strain sZJD-03 cell-free extract. The data represent the mean ± s.d. of biological duplicates.

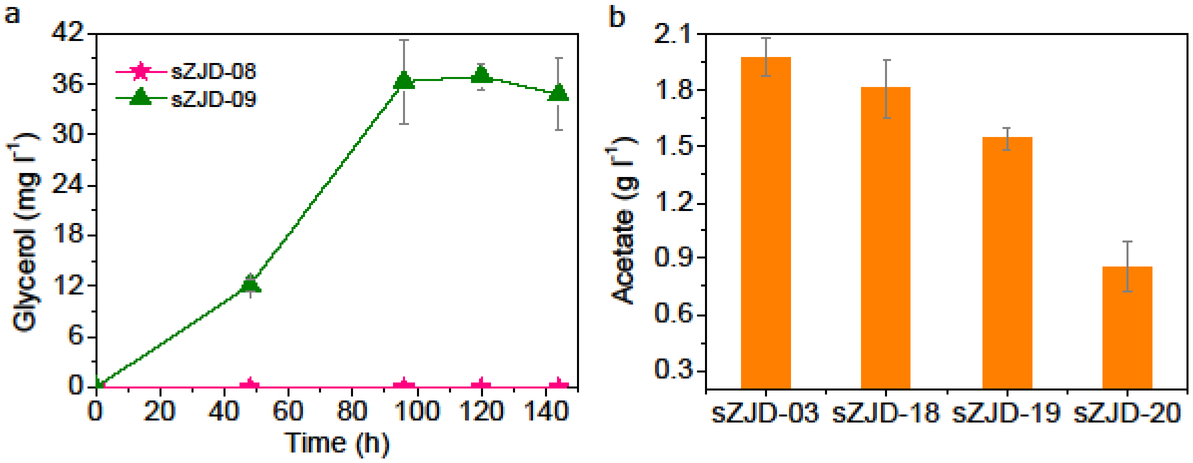

Supplementary Figure 3. Identification of promiscuous enzyme(s) for acetate accumulation**. (a)** Glycerol concentration in the medium of strain IMI076 harbouring the control plasmid (sZJD-08) and the POavPTAse containing plasmid (sZJD-09), respectively. To identify the promiscuous enzyme(s) in Pdc minus background yeast, Pdc^- *S. cerevisiae* IMI076 was used, because it could grow in excess glucose media without introducing exogenous acetyl-CoA producing pathway and be a comparable control strain. The data represent the mean ± s.d. of biological triplicates. Glycerol was produced by strain IMI076 containing the POavPTAse pathway in contrast to IMI076 with an empty plasmid. Glycerol 3-phosphate phosphatase (GPP) is responsible for catalyzing the conversion of glycerol 3-phosphate to glycerol. The structure of acetate-phosphate is similar to that of glycerol 3-phosphate, and we therefore suspected that GPP might be promiscuous involved in converting acetyl-phosphate to acetate. **(b)** Extracellular acetate concentration of Pdc^- strains with the POav/PTAse pathway carrying deleting in promiscuous phosphatase genes *GPP1* and *GPP2* in a synthetic medium containing 20 g l^{-1} glucose. sZJD-03: control, sZJD-18: *GPP1* single deletion, sZJD-19: *GPP2* single deletion, sZJD-20: *GPP1* and *GPP2* double deletion.

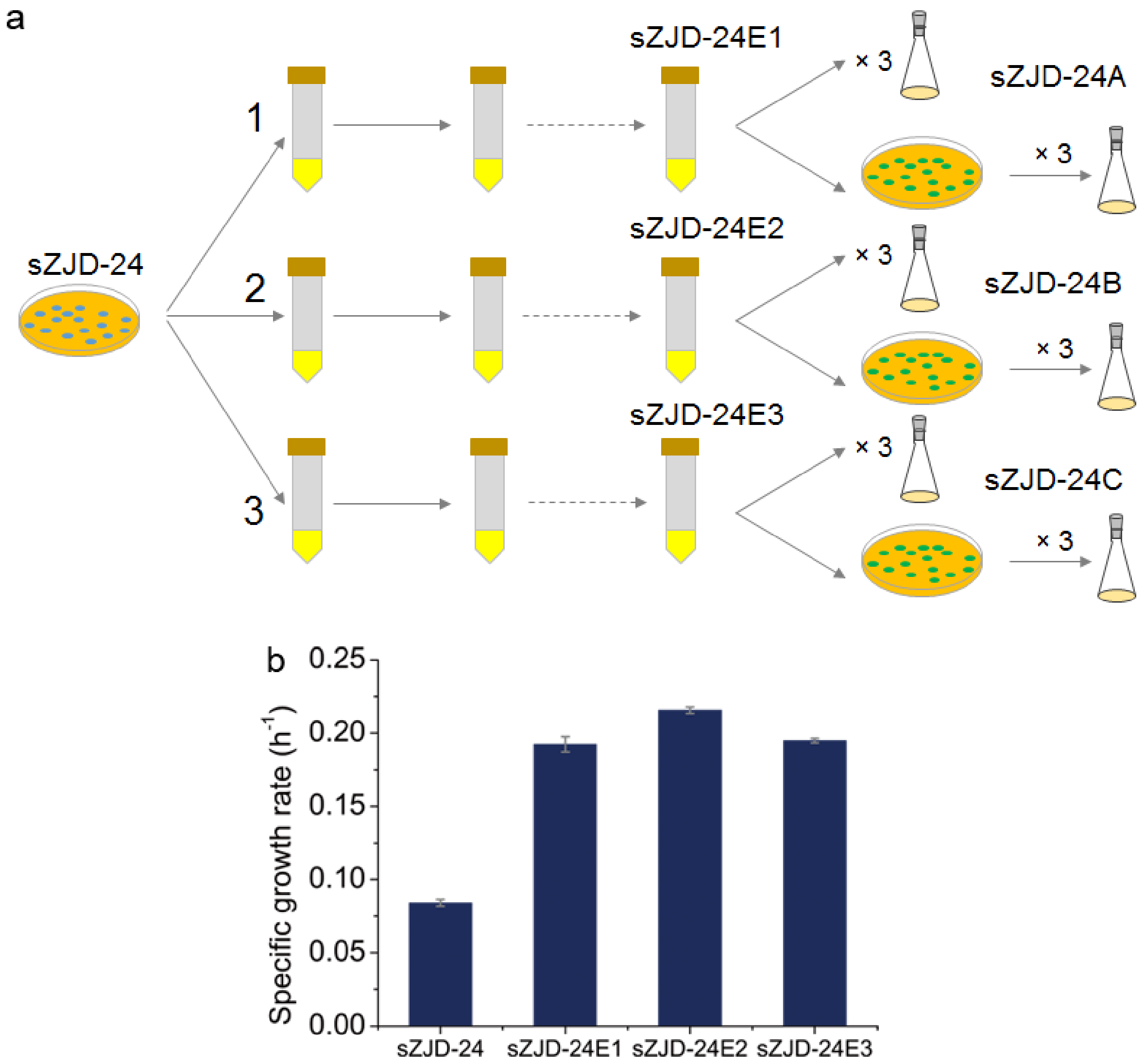

Supplementary Figure 4. ALE experimental setup and determination of specific growth rate. **(a)** Experimental procedure for ALE which generated three evolved populations (sZJD-24E1, sZJD-24E2 and sZJD-24E3). ×3 means 3 replicates and 3 clones were used for determine the specific growth rate. **(b)** The specific growth rate of three evolved populations and starting strain sZJD-24.

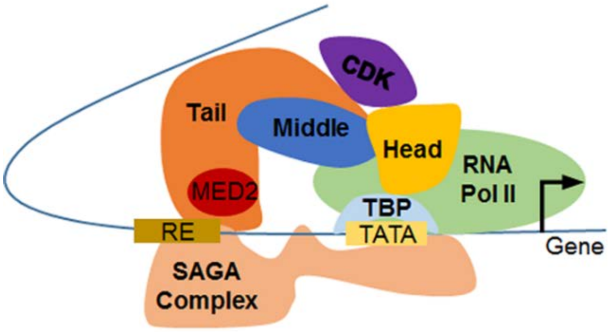

Supplementary Figure 5. Transcriptional activation mode of TATA-containing, SAGA-dependent genes, which requires tail module, SAGA complex, activators. Blue line indicates DNA.

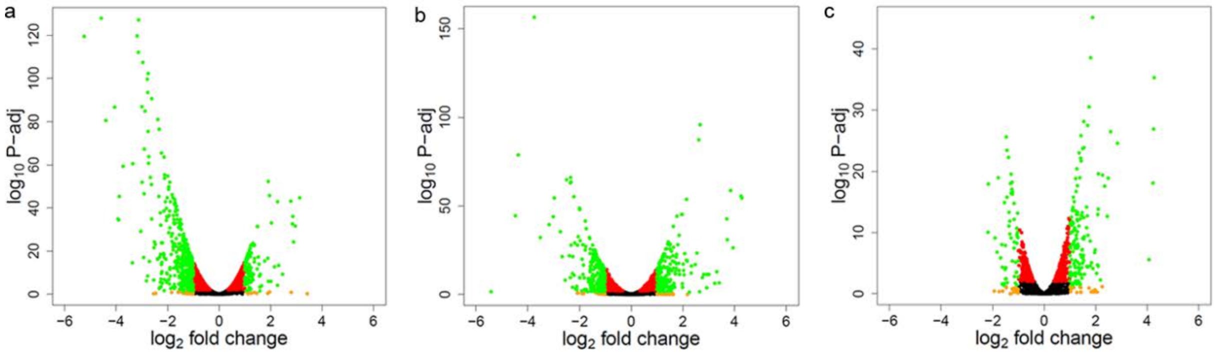

Supplementary Figure 6. Volcano plot of transcriptional changes of strains sZJD-28 (a), sZJD-27(b) and sZJD-26 (c) compared with strain sZJD-25. Green dots: padj< 0.01 & abs (log2 FC) > 1, red dots: padj< 0.01 & abs (log2 FC) ≤ 1, yellow dots: padj ≥ 0.01 & abs (log2 FC) > 1, black dots: padj ≥ 0.01 & abs (log2 FC) ≤ 1.

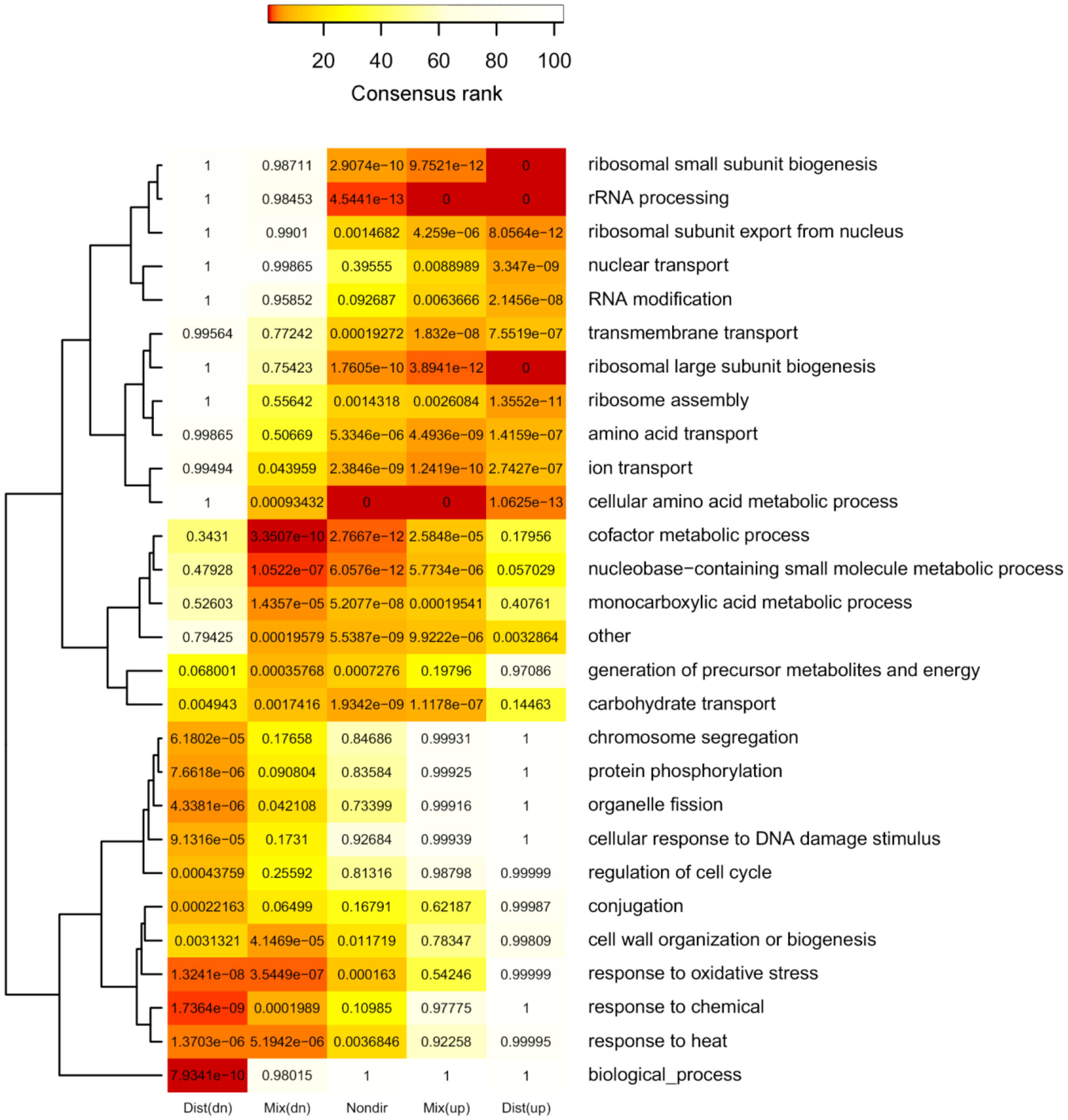

Supplementary Figure 7. Reporter GO term analysis of the transcription profiles of strain sZJD-27. The color key shows the rank of GO terms and the significance (p-value) of the GO term is included in each cell of the heatmap. GO terms that have a consensus rank ≤10 in any of the groups (distinct-directional down, mixed-directional down, non-directional change, mix-directional up and distinct-directional up) are shown in the heatmap.

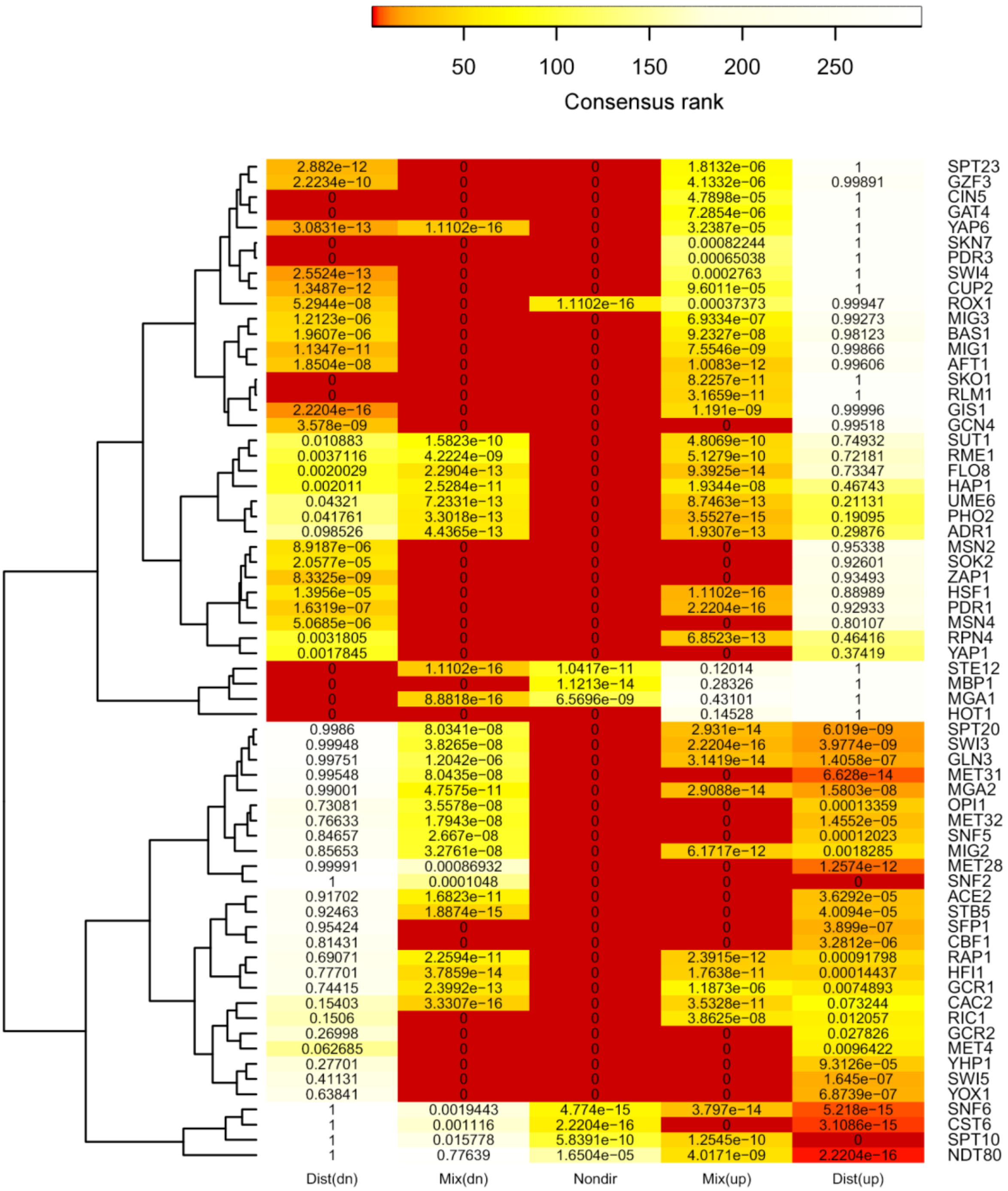

Supplementary Figure 8. Reporter transcription factors (TFs) analysis of strain sZJD-27 compared with control strain. The color key shows the rank of TFs and the significance (p-value) of the TF is included in each cell of the heatmap. TFs that have a consensus rank ≤5 in any of the groups (distinct-directional down, mixed-directional down, non-directional change, mix-directional up and distinct-directional up) are shown in the heatmap.

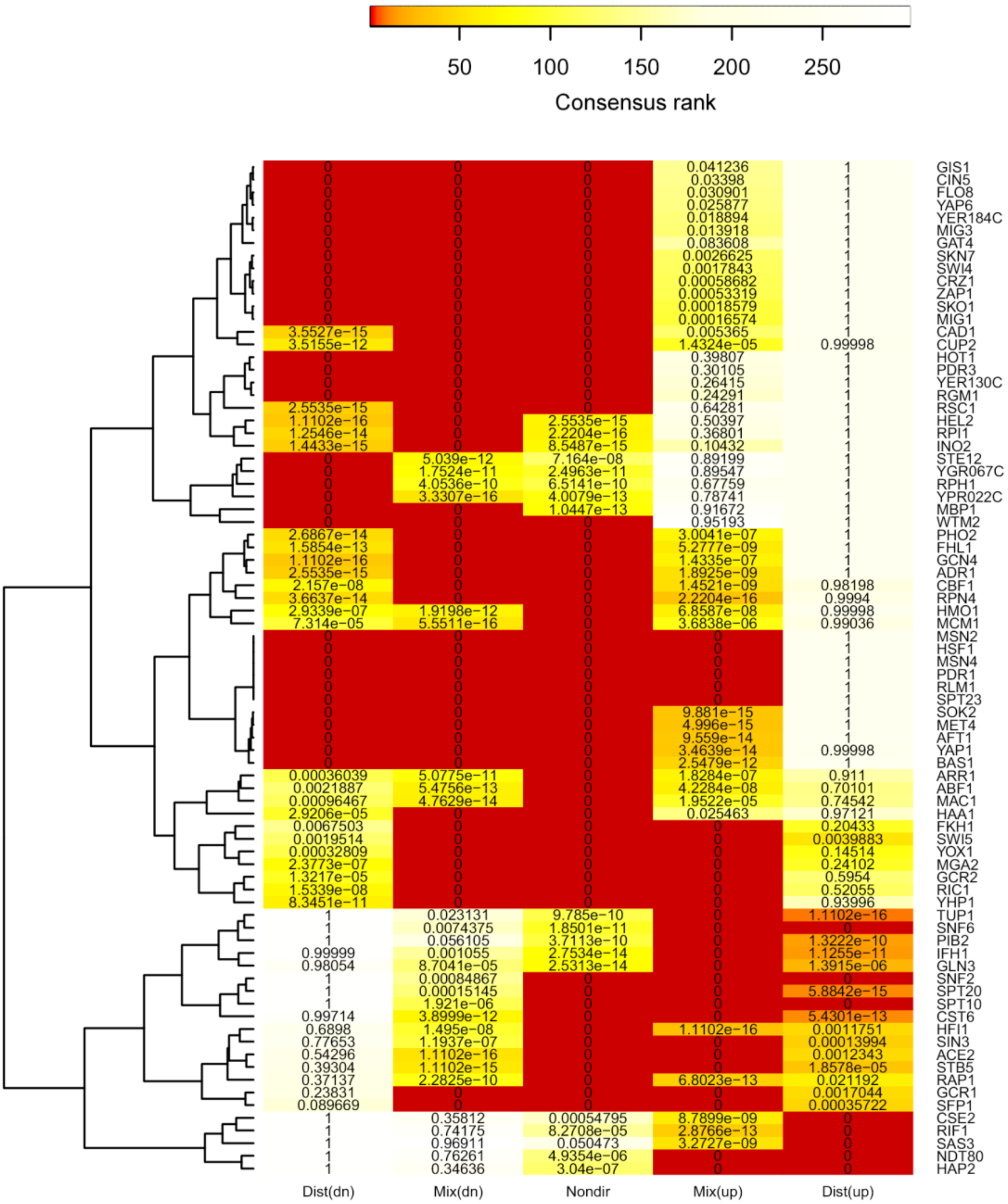

Supplementary Figure 9. Reporter transcription factors (TFs) analysis of strain sZJD-28 compared with control strain. The color key shows the rank of TFs and the significance (p-value) of the TF is included in each cell of the heatmap. TFs that have a consensus rank ≤5 in any of the groups (distinct-directional down, mixed-directional down, non-directional change, mix-directional up and distinct-directional up) are shown in the heatmap.

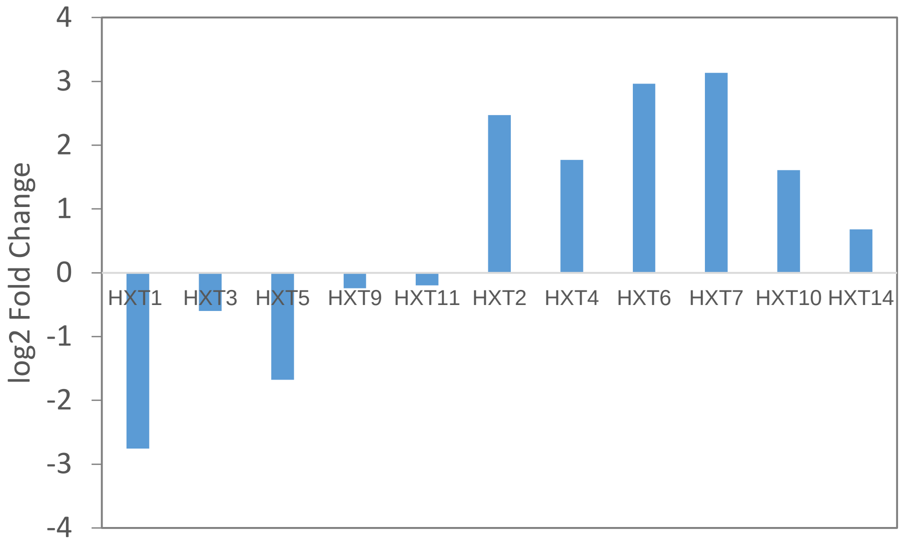

Supplementary Figure 10. Fold changes profiles of strain sZJD-28 compared with control strain sZJD-25.

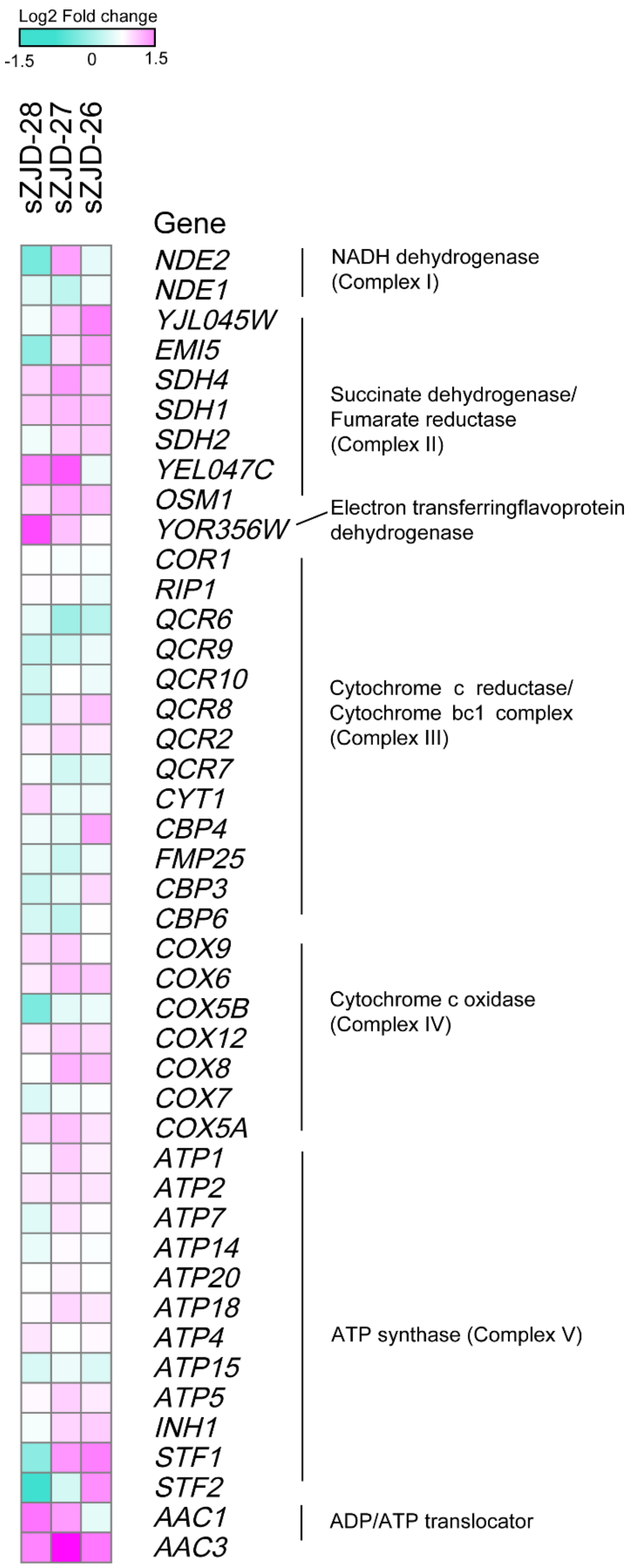

Supplementary Figure 11. Transcriptional levels of genes related to oxidative phosphorylation in sZJD-28, sZJD-27 and sZJD-26 compared with sZJD-25.

Supplementary Table 1. Strains used in this study

Strains	Genotype or characteristic	Source
CEN.PK YMZ-E1	MATa *ura3-52 his3-Δ1 pdc1Δ pdc5Δ pdc6Δ*	[1]
IMI076	MATa *pdc1Δ(-6,-2)*::*loxP pdc5Δ(-6,-2)*::*loxP pdc6Δ(-6,-2)*::*loxP ura3-52 MTH1-ΔT*	[2]
CEN.PK 113-11C	MATa *MAL2-8c SUC2 his3Δ1 ura3-52*	Kötter, University of Frankfurt, Germany
POspPTAse sZJD-01	CEN.PK YMZ-E1 with pZJD-01	This study
POlpPTAse sZJD-02	CEN.PK YMZ-E1 with pZJD-02	This study
POavPTAse sZJD-03	CEN.PK YMZ-E1 with pZJD-03	This study
Control sZJD-04	CEN.PK YMZ-E1 with pSP-GM1	This study
sZJD-08	IMI076 with pSP-GM1	This study
sZJD-09	IMI076 with pZJD-03	This study
sZJD-11	CEN.PK YMZ-E1(*acs2Δ*::*TEF1p-POav-ADH1t acs1Δ*::*TEF1p-PTAse-CYC1t*)	This study
sZJD-12	sZJD-11(pIST05)	This study
sZJD-13	sZJD-11(pYC1)	This study
sZJD-14	CEN.PK 113-11C(pIST05)	This study
HPY01	CEN.PK 113-11C(pYC1)	[3]
sZJD-15	CEN.PK YMZ-E1(*gpp1Δ*)	This study
sZJD-16	CEN.PK YMZ-E1(*gpp2Δ*)	This study
sZJD-17	CEN.PK YMZ-E1(*gpp1Δ gpp2Δ*)	This study
sZJD-18	sZJD-15(pZJD-03)	This study
sZJD-19	sZJD-16(pZJD-03)	This study
sZJD-20	sZJD-17(pZJD-03)	This study
sZJD-21	sZJD-11(*gpp1Δ*)	This study
sZJD-22	sZJD-11(*gpp2Δ*)	This study
sZJD-23	sZJD-11(*gpp1Δ gpp2Δ*)	This study
sZJD-24	sZJD-23(*XI-5*::*URA3 X-2*::*HIS3*)	This study
sZJD-24E1	Evolved sZJD-24	This study
sZJD-24E2	Evolved sZJD-24	This study
sZJD-24E3	Evolved sZJD-24	This study
sZJD-24A1	Clone from evolved sZJD-24E1	This study
sZJD-24A2	Clone from evolved sZJD-24E1	This study
sZJD-24A3	Clone from evolved sZJD-24E1	This study
sZJD-24B1	Clone from evolved sZJD-24E2	This study
sZJD-24B2	Clone from evolved sZJD-24E2	This study
sZJD-24B3	Clone from evolved sZJD-24E2	This study
sZJD-24C1	Clone from evolved sZJD-24E3	This study
sZJD-24C2	Clone from evolved sZJD-24E3	This study
sZJD-24C3	Clone from evolved sZJD-24E3	This study
sZJD-25	sZJD-23(*X-2*::*KanMX-TEF1p-Cas9-CYC1t*)	This study
sZJD-26	sZJD-25($GPD1^{W71*}$)	This study
sZJD-27	sZJD-25($MED2^{*432Y}$)	This study
sZJD-28	sZJD-25($MED1^{*432Y}GPD1^{W71*}$)	This study

Supplementary Table 2. Growth rate and ethanol yield on glucose of Crabtree negative and positive

Species	Yield: EtOH/Glu ($g\ g^{-1}$)	Growth rate (h^{-1})
*S. cerevisiae**	0.00	0.109
Kluyveromyces nonfermentans **	0.00	0.101
*Eremothecium sinecaudum A2***	0.00	0.117
*E. sinecaudum A1***	0.00	0.122
*K. Lactis A2***	0.00	0.255
*K. Marxianus B***	0.00	0.269
*K. Marxianus A1***	0.00	0.314
*K. Wickerhamii***	0.00	0.321
*Debaromyces vanrijiae A1***	0.00	0.347
*K. aestuarii***	0.00	0.429
*S. cerevisiae A2***	0.38	0.289

yeast

* Data from this study, **Data from previous work [4].

Supplementary Table 3. Mutations in evolved *S. cerevisiae* strains isolated from three populations

Strain	Mutations						
sZJD-24A1	*MED2*	*HXK2*	*EBP2*	*BEM3*	*ALG14*	*FAB1*	*MGA1*
	*432Y	Q195*	T415S	N673H	R20S	A620S	M274R
	TAG-TAT	CAA-TAA	ACC-AGC	AAT-CAT	AGG-AGT	GCT-TCT	ATG-AGG
sZJD-24A2	*MED2*	*HXK2*	*MCH2*	*NUD1*	*DIT2*	*GTO1*	
	*432Y	Q195*	A63T	G195G	V24F	F280Y	
	TAG-TAT	CAA-TAA	GCT-ACT	GGC-GGT	GTC-TTC	TTT-TAT	
sZJD-24B1	*MED3*	SIW14	*MHO1*	*TPS2*			
	L156*	W234*	T160K	Δ3bp			
	TTG-TAG	TGG-TGA	ACG-AAG	*2344-2346*			
sZJD-24B2	*MED3*	SIW14	*MHO1*	*AGP2*			
	L156*	W234*	T160K	C204Y			
	TTG-TAG	TGG-TGA	ACG-AAG	TGT-TAT			
sZJD-24C1	*MED2*	GPD1	DUN1				
	*432Y	W71*	T57A				
	TAG-TAT	TGG-TAG	ACA-GCA				
sZJD-24C2	*MED2*	GPD1					
	*432Y	W71*					
	TAG-TAT	TGG-TAG					
sZJD-24C3	*MED2*	GPD1					
	*432Y	W71*					
	TAG-TAT	TGG-TAG					

Supplementary Table 4. Plasmids used in this study

Plasmids	Genotype or characteristic	Source
pSP-GM1	2 µm, *AmpR*, *URA3*, TEF1p,ADH1t, PGK1p, CYC1t	[5]
pSP-GM2	2 µm, *AmpR*, *URA3*, TEF1p, CYC1t, PGK1p, ADH1t	[5]
pZJD-01	pSP-GM1-(TEF1p-POsp-ADH1t, PGK1p-PTAse-CYC1t)	This study
pZJD-02	pSP-GM1-(TEF1p-POlp-ADH1t, PGK1p-PTAse-CYC1t)	This study
pZJD-03	pSP-GM1-(TEF1p-POav-ADH1t, PGK1p-PTAse-CYC1t)	This study
pIST05	pSP-GM1-(TEF1p-FarnSYNcj-ADH1t, PGK1p-tHMG1-CYC1t)	[6]
pYC1	pSP-GM2-(TEF1p-MCRca-CYC1t)	[3]
pUG6	loxP-pAgTEF1-kanMX-tAgTEF1-loxP, AmpR, ori	[7]
pCfB2312	CEN/ARS, KanMX, AmpR, TEF1p, Cas9, CYC1t	[8]

Supplementary References

1 Zhang, Y. *et al.* Adaptive mutations in sugar metabolism restore growth on glucose in a pyruvate decarboxylase negative yeast strain. *Microb. Cell Fact.* **14**, 116, doi:10.1186/s12934-015-0305-6 (2015).

2 Oud, B. *et al.* An internal deletion in *MTH1* enables growth on glucose of pyruvate-decarboxylase negative, non-fermentative *Saccharomyces cerevisiae*. *Microb. Cell Fact.* **11**, 131, doi:10.1186/1475-2859-11-131 (2012).

3 Chen, Y., Bao, J. C., Kim, I. K., Siewers, V. & Nielsen, J. Coupled incremental precursor and co-factor supply improves 3-hydroxypropionic acid production in *Saccharomyces cerevisiae*. *Metab. Eng.* **22**, 104-109, doi:10.1016/j.ymben.2014.01.005 (2014).

4 Hagman, A., Sall, T., Compagno, C. & Piskur, J. Yeast "Make-Accumulate-Consume" Life Strategy Evolved as a Multi-Step Process That Predates the Whole Genome Duplication. *Plos One* **8**, e68734, doi:ARTN e68734 10.1371/journal.pone.0068734 (2013).

5 Chen, Y., Partow, S., Scalcinati, G., Siewers, V. & Nielsen, J. Enhancing the copy number of episomal plasmids in *Saccharomyces cerevisiae* for improved protein production. *FEMS Yeast Res.* **12**, 598-607, doi:10.1111/j.1567-1364.2012.00809.x (2012).

6 Tippmann, S., Scalcinati, G., Siewers, V. & Nielsen, J. Production of farnesene and santalene by *Saccharomyces cerevisiae* using fed-batch cultivations with RQ-controlled feed. *Biotechnol. Bioeng.* **113**, 72-81, doi:10.1002/bit.25683 (2016).

7 Guldener, U., Heck, S., Fiedler, T., Beinhauer, J. & Hegemann, J. H. A new efficient gene disruption cassette for repeated use in budding yeast. *Nucleic Acids Res.* **24**, 2519-2524, doi:DOI 10.1093/nar/24.13.2519 (1996).

8 Stovicek, V., Borodina, I. & Forster, J. CRISPR–Cas system enables fast and simple genome editing of industrial *Saccharomyces cerevisiae* strains. *Metab. Eng. Commun.* **2**, 13-22 (2015).
