# Supplementary material for: Global rewiring of cellular metabolism renders Saccharomyces cerevisiae Crabtree negative
Source: Nat Commun. 2018 Aug 3;9:3059. doi: 10.1038/s41467-018-05409-9 (PMC6076296; doi:10.1038/s41467-018-05409-9)
Supplement: Supplementary file 3 — Description of Additional Supplementary Files [file 41467_2018_5409_MOESM3_ESM.pdf]

Descriptions of Additional Supplementary Files

File Name: Supplementary Dataset 1
Description: GO Slim Mapper_shared 114 TATA-containing genes

File Name: Supplementary Dataset 2
Description: Codon optimized genes' sequences and primers used in this study
